# A Novel Nomogram for Predicting Meningioma Grade Based on Radiomics Features and Clinical Characteristics

**DOI:** 10.1007/s11596-026-00199-4

**Published:** 2026-05-04

**Authors:** Peng-fei Yan, Bao-ping Zheng, Ye Yuan, Zhen Zhao, Hao-jun Shi, Dong-xiao Yao

**Affiliations:** 1 Department of Neurosurgery, Union Hospital, Tongji Medical College, Huazhong University of Science and Technology, Wuhan, 430022 China; 2 Department of Wound Repair and Vascular Surgery II, Liyuan Hospital, Tongji Medical College, Huazhong University of Science and Technology, Wuhan, 430060 China; 3 Department of Radiology, Union Hospital, Tongji Medical College, Huazhong University of Science and Technology, Wuhan, 430022 China

**Keywords:** Meningioma, Magnetic resonance imaging (MRI), Radiomics, Tumor grading, Machine learning, Nomogram, Logistic regression

## Abstract

**Objective:**

This study aimed to develop a predictive model utilizing radiomics features and clinical characteristics to accurately differentiate low-grade (WHO grade I) from high-grade (WHO grade II/III) meningiomas preoperatively, thereby improving treatment planning and prognosis.

**Methods:**

A retrospective analysis of 288 meningioma cases (191 low-grade and 97 high-grade) confirmed by histopathology was conducted. Radiomics features were extracted from contrast-enhanced T1-weighted MRI (CE-T1WI) using the pyradiomics package, followed by feature selection via LASSO regression. Predictive models (logistic regression, decision tree, support vector machine [SVM], adaptive boosting) were evaluated. Clinical variables (peritumoral edema index and monocyte count) were integrated to try to improve the predictive performance. Model efficacy was assessed using receiver operating characteristic (ROC) curves, calibration plots, and decision curve analysis.

**Results:**

Four key radiomics features were identified as significant discriminators of tumor grade. The logistic regression model demonstrated superior predictive performance over decision trees, SVMs, and adaptive boosting methods. The inclusion of the peritumoral edema index and monocyte count increased the AUC to 0.801 (95% CI 0.753–0.869) in the training set. However, in the validation set, the radiomics model achieved the best performance, with an AUC of 0.770 (95% CI 0.670–0.869).

**Conclusions:**

The radiomics-based model effectively predicts high-grade meningioma and demonstrates superior performance compared to the clinical and combined models. This study advances the precision of meningioma grading, offering significant implications for treatment planning and patient management.

**Supplementary Information:**

The online version contains supplementary material available at 10.1007/s11596-026-00199-4.

## Introduction

Meningiomas, originating from arachnoid cap cells situated in the dura mater, constitute the most prevalent primary intracranial neoplasms. Recent data from the Central Brain Tumor Registry of the United States have documented an annual increase in incidence of 0.9% [1, 2]. Notably, approximately 80% of meningiomas are grade I according to the World Health Organization (WHO), indicative of benign pathology, whereas the remaining are categorized as atypical (grade II) or malignant (grade III) [3]. Compared with their low-grade counterparts, those with high-grade meningiomas (II/III) have significantly inferior clinical outcomes [4, 5]. While patients with benign meningiomas have a five-year survival rate of 92.1% [6], this figure drops to 77.6% for those with atypical meningiomas [7] and plummets to 45% for those diagnosed with malignant variants [8]. Thus, early and precise stratification of meningioma grade is imperative for guiding therapeutic decisions and reducing mortality.

Radiomics represents an emerging field characterized by the high-throughput mining of extensive data from medical imagery, facilitating tumor delineation, feature extraction, and predictive model development [9]. This field boasts two principal advantages over traditional radiological methods: First, radiomics-derived features provide comprehensive insights into the tumor phenotype and microenvironment, surpassing conventional data extraction techniques. Second, the high-dimensional data encapsulated within these features elucidate the complexity of the disease by capturing intratumoral heterogeneity [10]. Within the swiftly advancing realm of neuroscience, radiomics is increasingly recognized as a valuable tool for tailored oncological therapies [11, 12]. Recent research has indicated its expanding application in tumor classification, monitoring treatment efficacy, and prognostication [13–15].

This investigation used radiomics to stratify meningioma diagnosis. Our objective was to construct a composite model that integrates radiomics-derived tumor-region features with clinical parameters to distinguish meningiomas of varying WHO grades. The findings of this study have the potential to advance the stratified management of meningiomas.

## Materials and Methods

### Study Design and Participants

**Fig. 1:**
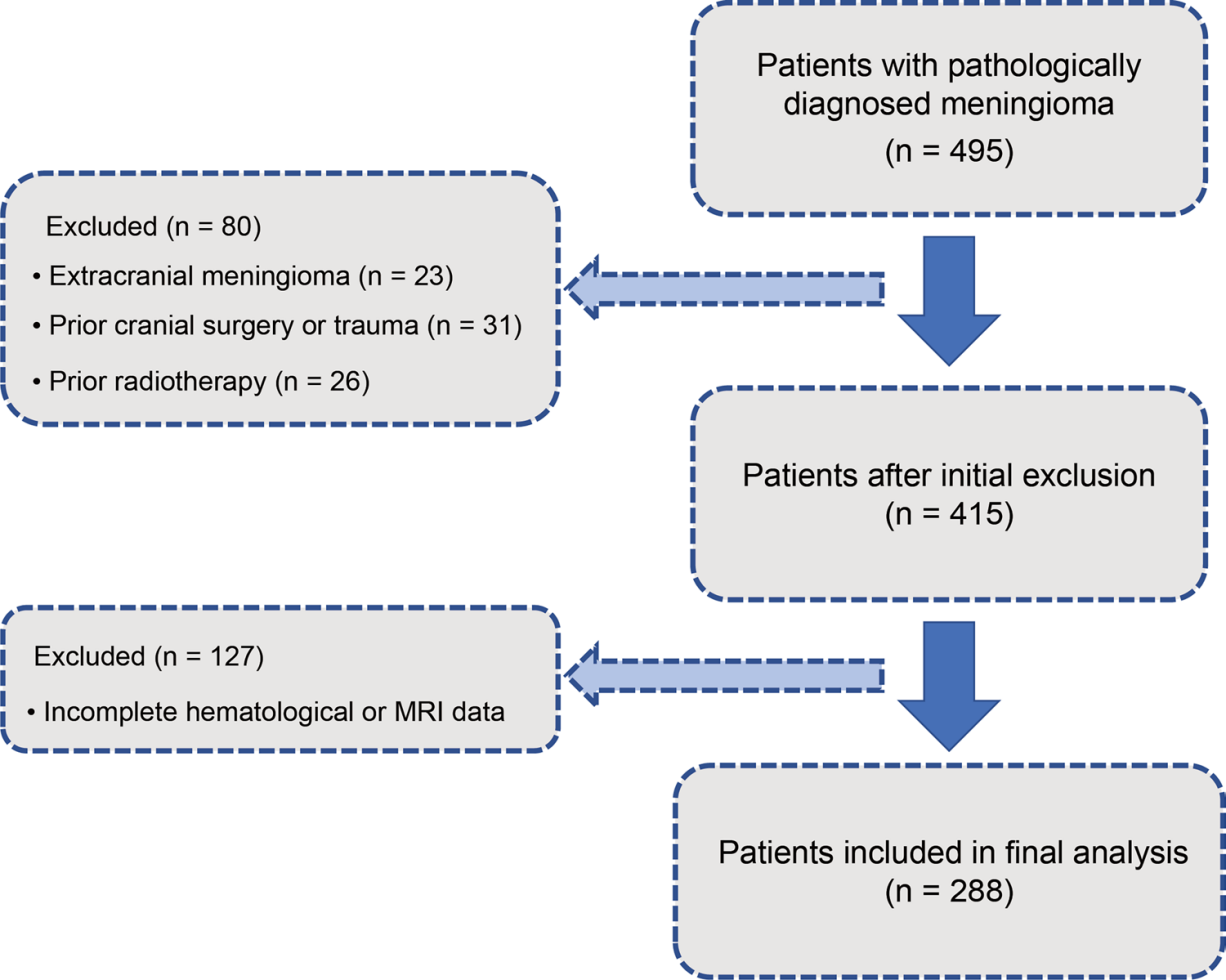
Flowchart of patient selection for this study

The retrospective study received a waiver for written consent from Union Hospital, Tongji Medical College, Huazhong University of Science and Technology (China). With the approval of the institutional review board (approval No. 2026-0024), we reviewed 495 patients who were diagnosed with meningioma via postoperative pathology from January 2015 to December 2019. The histopathological classification adhered to the 2016 WHO criteria. The exclusion criteria included patients with extracranial meningiomas, patients who underwent prior cranial surgery or trauma, and patients who underwent radiotherapy. We compiled medical records, preoperative laboratory tests, and magnetic resonance imaging (MRI) reports from the hospital’s documentation system. An additional 127 patients were excluded because of incomplete hematological or MRI data. Consequently, 288 patients constituted the cohort for our final analysis (Fig. 1). In this study, MRI was performed using 3.0 T (Siemens Trio, Erlangen, Germany) and 1.5 T (Siemens Avanto, Erlangen, Germany) MR scanners equipped with eight-channel head radiofrequency coils. The key acquisition parameters for the sequences used in this analysis were as follows: a field of view of 230 × 230 mm, a matrix size of 512 × 512, a slice thickness of 5 mm, and a flip angle of 90°. The repetition times (TRs)/echo times (TEs) were 500/8.4 ms for the T1-weighted imaging (T1WI) and 9,000/105 ms for the fluid-attenuated inversion recovery (FLAIR) sequences, respectively. Contrast-enhanced T1WI (CE-T1WI) was performed following intravenous administration of 0.2 mL/kg gadopentetate dimeglumine [16, 17]. All the images were archived and accessed via a picture archiving and communication system (PACS).

### MRI and Image Analysis

For image analysis, we employed CE-T1WI and FLAIR sequences, with segmentation performed via ITK-SNAP software (version 3.8.0, http://www.itksnap.org) [18]. CE-T1WI facilitated visualization of the brain–tumor interface, whereas FLAIR delineated the boundary of peritumoral edema. Segmentation of meningiomas on CE-T1WI was performed manually by a neurology researcher with six years of experience (Zhen Zhao), and peritumoral edema outlines on FLAIR were similarly drawn by another researcher with three years of experience (Bao-ping Zheng). A senior neurologist with ten years of experience (Peng-fei Yan) reviewed all the delineations for consensus. The FLAIR images included the tumor area; therefore, to determine the peritumoral edema volume (PEV), we deducted the tumor volume from the total FLAIR contour area. We defined the peritumoral edema index (PEI) as the ratio of the PEV to the tumor volume. Researchers were blinded to the patients’ clinical information and histological grades during this phase.

### Radiomics and Modeling

Radiomic feature extraction was performed using the open-source Python package, PyRadiomics (version 3.0.0, http://github.com/AIM-Harvard/pyradiomics) [19]. In pursuit of clinically significant features, we generated 10 supplementary images per case by applying Laplacian of Gaussian (LoG) or wavelet filters to the original images. This process yielded 1,015 radiomic features from the regions of interest in each CE-T1WI image modality, including 14 shape, 306 first-order, and 695 texture features.

To mitigate overfitting and prioritize feature analysis given the extensive array of radiomic features obtained, we implemented a systematic selection process. Initially, any missing values within the feature data were imputed using the mean substitution method. We subsequently standardized the features using Z-score normalization, thereby transforming them to a distribution with a mean of 0 and a standard deviation of 1. In the subsequent phase, the dataset was randomly divided into training and test cohorts at a 7:3 ratio. Finally, we applied the least absolute shrinkage and selection operator (LASSO) regression for dimensionality reduction within the training set, using tenfold cross-validation to extract features with nonzero coefficients [20].

Following the initial screening, the retained radiomic features were integrated into various predictive models using logistic regression (LR), decision trees (DT), support vector machines (SVMs), and adaptive boosting techniques. We assessed the performance of these classifiers by constructing receiver operating characteristic (ROC) curves. A classifier with superior efficacy was then used to develop both clinical and combined predictive models. The performance metrics considered included sensitivity, specificity, accuracy, and the area under the curve (AUC). A predictive nomogram was subsequently constructed, and its predictive accuracy was validated by means of calibration and decision curve analyses.

### Statistical Analysis

Statistical analysis was performed using Python version 3.7.1 (https://www.python.org), R version 3.4.1 (http://www.r-project.org), and EmpowerStats software (http://empowerstats.com). Python facilitated the extraction and refinement of radiomic features, whereas R was employed for the development and assessment of the predictive models. EmpowerStats aided in the comparative analysis of variables across different groups. Normally distributed continuous variables are reported as mean ± standard deviation (SD), and nonnormally distributed variables are reported as median with range (maximum, minimum). Categorical variables are denoted as percentages. The independent samples *t*-test was used to compare continuous variables between the low-grade and high-grade meningioma groups, and the chi-square test was used for categorical variables. A *P* value less than 0.05 was considered to indicate statistical significance.

## Results

### Baseline Characteristics of Participants

Table 1 summarizes the fundamental clinical characteristics, laboratory parameters, and clinicopathological findings of the study cohort. Among the 288 patients assessed, 191 had low-grade meningiomas, whereas 97 had high-grade meningiomas. Females constituted 72.57% of the participants, with a mean age of 53.67 ± 10.22 years. Analysis of the MRI scans revealed 121 convexity meningiomas, 76 parasagittal/falx, 43 cranial base, and 48 posterior fossa meningiomas. Noteworthy differences between the low-grade and high-grade groups were evident in tumor volume, peritumoral edema, PEI, monocyte count, and the monocyte-to-lymphocyte ratio (MLR). Conversely, no significant disparities were detected in the other examined variables.

**Table 1:** Baseline characteristics of patients

Variables	Low grade	High grade	Total	*P* value
n	191	97	288	
Sex				0.074
Male	46 (24.08%)	33 (34.02%)	79 (27.43%)	
Female	145 (75.92%)	64 (65.98%)	209 (72.57%)	
Age/year (mean ± SD)	53.60 ± 9.75	53.79 ± 11.16	53.67 ± 10.22	0.881
Location				0.219
Convexity	72 (37.70%)	49 (50.52%)	121 (42.01%)	
Parasagittal/falx	55 (28.80%)	21 (21.65%)	76 (26.39%)	
Cranial base	30 (15.71%)	13 (13.40%)	43 (14.93%)	
Posterior fossa	34 (17.80%)	14 (14.43%)	48 (16.67%)	
Tumor volume/mL (median [min–max])	25.19 (3.05–88.32)	33.19 (3.49–175.64)	33.57 (3.05–175.64)	<0.001
Peritumoral edema volume/mL (median [min–max])	17.77 (2.22–149.05)	50.37 (2.52–235.80)	45.33 (2.22–235.80)	<0.001
PEI (median [min–max])	0.81 (0.06–9.61)	1.40 (0.07–30.09)	1.80 (0.06–30.09)	0.001
Laboratory test (mean ± SD)				
Leukocyte (× 10^9^/L)	7.41 ± 3.90	7.55 ± 4.09	7.46 ± 3.96	0.780
Erythrocyte (× 10^9^/L)	4.20 ± 0.51	4.22 ± 0.58	4.21 ± 0.54	0.735
Platelet (× 10^9^/L)	192.43 ± 60.36	199.20 ± 59.57	194.71 ± 60.07	0.367
Neutrophil (× 10^9^/L)	5.41 ± 4.14	5.40 ± 4.15	5.40 ± 4.14	0.991
Lymphocyte (× 10^9^/L)	1.48 ± 0.67	1.53 ± 0.70	1.50 ± 0.68	0.545
Monocyte (× 10^9^/L)	0.40 ± 0.23	0.48 ± 0.23	0.43 ± 0.23	0.007
Albumin (g/L)	39.38 ± 5.40	38.27 ± 5.18	39.00 ± 5.34	0.098
NLR (median [min–max])	2.22 (0.59–37.09)	2.40 (0.75–57.23)	6.22 (0.59–57.23)	0.809
dNLR (median [min–max])	1.64 (0.48–23.53)	1.75 (0.46–23.37)	3.71 (0.46–23.53)	0.236
PLR (median [min–max])	129.23 (41.67–623.91)	140.25 (41.51–518.18)	159.89 (41.51–623.91)	0.566
MLR (median [min–max])	0.24 (0.01–1.98)	0.28 (0.10–2.86)	0.37 (0.01–2.86)	0.021
NPR (median [min–max])	0.02 (0.01–0.17)	0.02 (0.01–0.16)	0.03 (0.01–0.17)	0.880
PNI (median [min–max])	47.85 (20.85–62.65)	46.90 (28.75–59.80)	46.50 (20.85–62.65)	0.338
SII (median [min–max])	442.22 (101.18–10,914.27)	536.25 (107.41–6,906.14)	1103.74 (101.18–1,0914.27)	0.423

### Selection of Radiomics Features

**Fig. 2:**
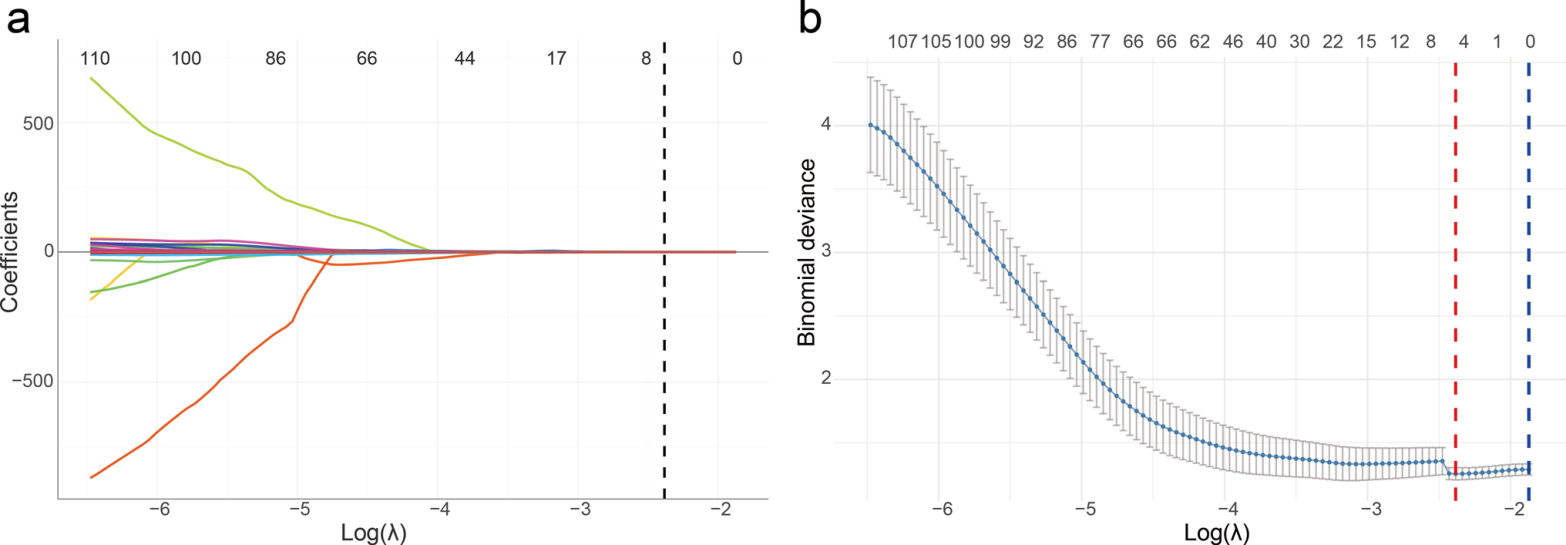
**a** LASSO coefficient profile plot of the features. The ordinate was the LASSO coefficient, the lower abscissa was the log(λ) value, and the upper abscissa was the number of nonzero coefficients. As the log(λ) value gradually increased, the LASSO coefficient gradually decreased to 0. **b** Selection in the LASSO model by minimum criteria using ten-fold cross-validation. The lower abscissa was the log(λ) value. The upper abscissa showed the number of LASSO features corresponding to the log(λ) value after dimensionality reduction. The left-dotted line represented the optimal values using the minimum criteria, and the right-dashed line represented one standard error of the minimum criteria

From the enhanced T1-weighted images, a comprehensive set of 1,015 radiomic features was initially extracted. Following rigorous standardization via Z-score normalization, discriminative analysis through feature optimization with LASSO regression coupled with ten-fold cross-validation, four salient radiomic features within the tumor region were identified. These features hold significant potential in grading meningiomas. The details of these features are depicted in Fig. 2 and listed in Table S1.

### Analysis of Predictive Efficacy

Assessment of the predictive models, as indicated in Table 2, revealed that the SVM model performed better in the training cohort. In contrast, the LR model performed best in the test cohort. A cumulative appraisal based on the AUC metric showed the LR model’s superior predictive performance, with AUCs of 0.736 in the training group and 0.770 in the test group.

**Table 2:** Predictive effectiveness of four classifiers

Model	Training set				Test set			
	AUC (95%CI)	Sensitivity (%)	Specificity (%)	Accuracy (%)	AUC (95%CI)	Sensitivity (%)	Specificity (%)	Accuracy (%)
LR	0.736 (0.659–0.812)	80.5	56.7	72.5	0.770 (0.670–0.869)	74.1	73.3	73.9
DT	0.715 (0.646–0.784)	85.0	53.7	74.5	0.617 (0.503–0.730)	69.0	53.3	63.6
SVM	0.787 (0.717–0.858)	82.7	61.2	76.5	0.633 (0.499–0.767)	70.7	66.7	69.3
AdaBoost	0.746 (0.669–0.822)	51.1	79.1	60.5	0.706 (0.596–0.816)	51.7	90.0	64.8

### Comparative Assessment of Predictive Models

**Fig. 3:**
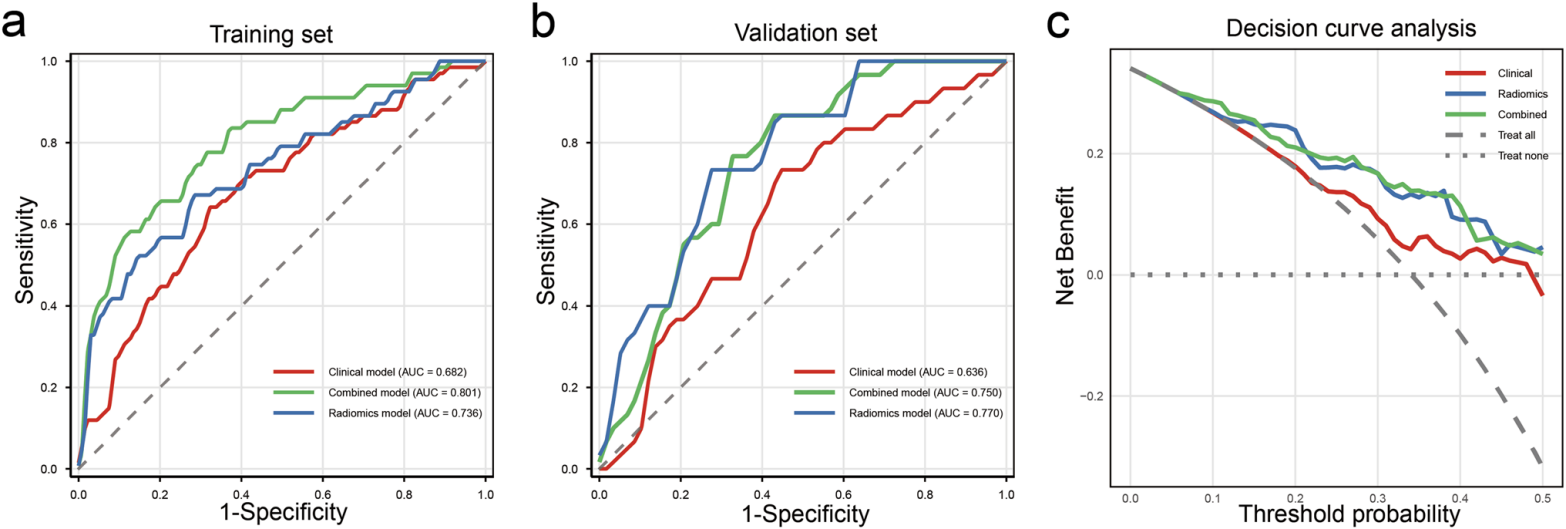
**a–c** ROC curves of the radiomics, clinical, and combined models based on the LR classifier in the training group (**a**) and test group (**b**) and decision curves of different models (**c**). Different colored lines represent the corresponding predictive models. The combined model achieved the highest AUC in the training cohort, whereas the radiomics model demonstrated superior performance in the test cohort. Decision curve analysis revealed comparable net benefits between the combined model and the radiomics-only model across the full range of threshold probabilities

A clinical model was developed by integrating monocyte counts and the PEI with the LR algorithm. The selection of LR as the foundational algorithm was based on its optimal performance among the tested radiomics models and, crucially, its superior clinical interpretability. Therefore, model interpretability was a key criterion in selecting the final model. LR provides inherent clinical interpretability, as its coefficients directly quantify the strength of association between predictors and the outcome, making it highly suitable for developing a clinically translatable prediction tool. We subsequently combined clinical parameters with radiomic features, aiming to incorporate multidimensional data to refine the LR-based radiomic clinical model. The ROC curves indicating the predictive accuracy of each model are depicted (Fig. 3a, b), with a corresponding efficiency comparison presented in Table 3. The enhanced predictive accuracy of the combined radiomics-clinical model for histopathological grading of meningiomas was confirmed, with an AUC of 0.801 in the training cohort. However, unexpectedly, its performance in the validation cohort was inferior to that of the radiomics-only model. Decision curve analysis demonstrated that the combined model and the radiomics-only model exhibited comparable net benefits across the full range of threshold probabilities, suggesting that the radiomics model alone already possesses good clinical utility (Fig. 3c).

**Table 3:** Predictive effectiveness of different models

	Training set				Test set			
Model	AUC (95%CI)	Sensitivity (%)	Specificity (%)	Accuracy (%)	AUC (95%CI)	Sensitivity (%)	Specificity (%)	Accuracy (%)
Clinical model	0.682 (0.603–0.761)	64.2	68.4	67.0	0.636 (0.513–0.758)	73.3	56.9	62.5
Radiomics model	0.736 (0.659–0.812)	67.2	72.2	70.5	0.770 (0.670–0.869)	73.3	74.1	73.9
Combined model	0.801 (0.734–0.868)	77.6	69.2	72.0	0.750 (0.648–0.852)	76.7	69.0	71.6

### Construction and Validation of a Predictive Nomogram

**Fig. 4:**
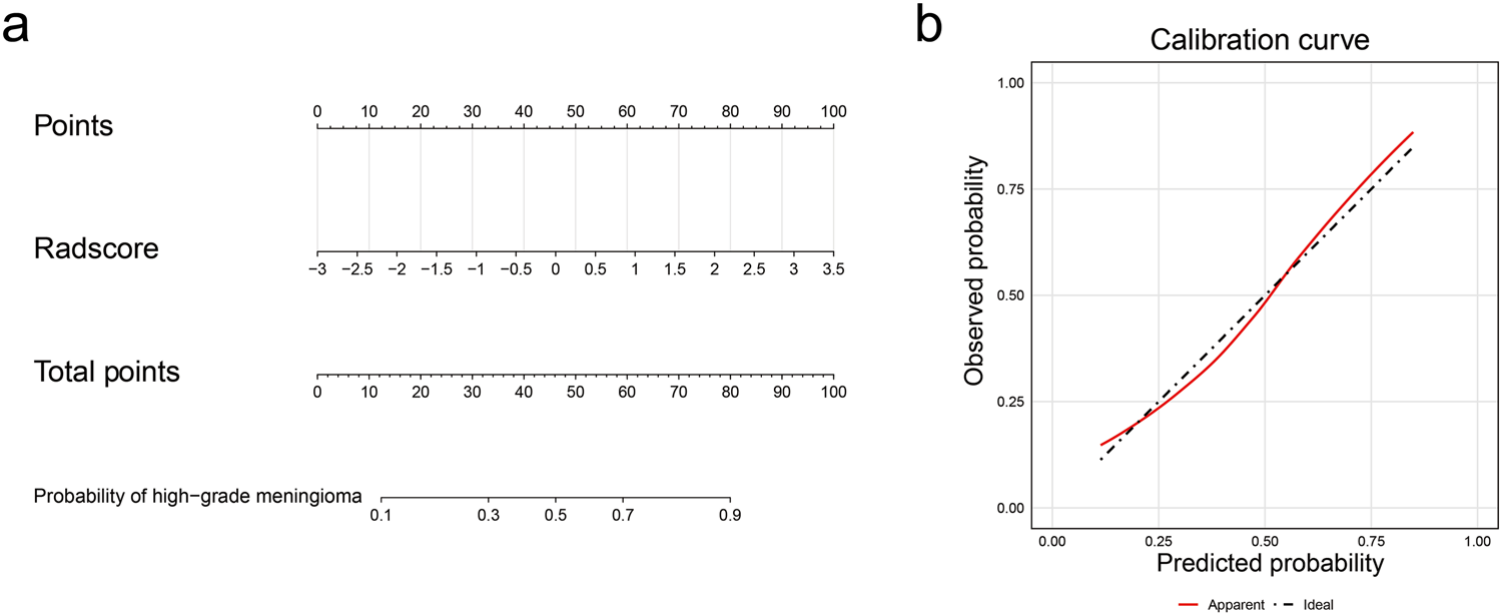
**a** Nomogram for predicting meningioma grade. **b** A calibration curve of the nomogram predictive model. The models were calibrated in terms of consistency. The abscissa is the predicted value, the ordinate is the actual value, and the black line represents the prediction by an ideal model

A nomogram was constructed utilizing the LR algorithm to facilitate individualized prediction of meningioma grade (Fig. 4a). The nomogram incorporated the radiomics signature score, which ranged from −3 to 3.5 and corresponded to a point range of 0 to 100, as the sole predictor for total score calculation. The relative length of the variable axis in the nomogram graphically represents its weight in the predictive model. For clinical application, a total point score above 65 corresponds to a predicted risk of high-grade meningioma exceeding 90%, providing a useful threshold for identifying high-risk patients. The total score is directly proportional to the likelihood of a WHO grade exceeding I. The predictive accuracy of the nomogram was extensively assessed through 1,000 bootstrap resamples, which revealed a near-perfect 45-degree calibration line in the calibration plot (Fig. 4b). These findings indicate that the nomogram’s predictions are well aligned with the actual clinical outcomes, confirming its reliable calibration.

## Discussion

This study aimed to construct a predictive model that utilizes preoperative imaging data and hematological indicators to accurately predict the pathological classification of meningiomas. The selection of radiomic features from CE-T1WI through LASSO regression proved to be a significant discriminative factor for high-grade meningiomas. Notably, the logistic regression model distinguished itself with the highest AUC in the ROC curve analysis, signifying its superior classification capability. By integrating clinical parameters with radiomic features into a composite model, the AUC reached 0.801 in the training cohort; however, this integration did not improve the AUC in the validation cohort. We consider that this may be due to the increased complexity of the integrated model, which, when faced with a relatively small training set, tends to memorize noise rather than learn the true underlying patterns, thereby leading to decreased performance in the test set. Furthermore, we converted the radiomic model with the best predictive performance into a user-friendly nomogram, as shown in Fig. 4, designed to enhance clinical utility by offering a practical method for estimating the WHO grades of meningioma, thus supporting more nuanced clinical decision-making.

Meningiomas are the most common primary intracranial tumors in adults and are generally characterized by slow growth. A minority of atypical and malignant meningiomas present a significant risk of aggressive behavior and recurrence [21]. Notably, some tumors initially classified as WHO grade I can exhibit invasive tendencies, highlighting the need for vigilant management [22]. The accurate identification of high-risk meningiomas is vital for appropriate prognostic assessment and the tailoring of therapeutic approaches.

Radiomics represents a cutting-edge application of artificial intelligence in image analysis, leveraging the extraction of extensive features from medical imaging to decipher tumor heterogeneity and enhance the interpretation of clinical data [10, 23]. Coroller et al.’s model demonstrated considerable accuracy for meningioma classification, which was further improved by incorporating semantic features [24]. Comparative studies, such as that by Hu et al., highlighted the benefits of combining traditional MRI with other imaging modalities, which enhanced the model’s predictive performance [25]. Hale et al. used machine learning to create a model that accurately predicted atypical meningioma grades [26]. These studies underscore the growing potential of computational analytics in refining diagnostic precision and facilitating personalized treatment plans. Despite their innovative contributions, these studies did not culminate in the creation of a practical tool for clinical decision-making. Our study addresses this issue by proposing a model that combines radiomic and clinical data to predict meningioma grade effectively. Although the predictive performance of our model is slightly lower than that of some reported methods [27], this discrepancy can likely be attributed to differences in MR imaging sequences and data processing methods, underscoring the need for standardization in imaging and analysis protocols. Notably, the predictive model and nomogram presented here are exploratory. Their integration into routine clinical workflows necessitates prospective validation in larger, multicenter cohorts to confirm their generalizability and clinical utility.

Our research revealed three critical insights. Primarily, the probability of a meningioma being high-grade was directly associated with its size, which is consistent with the findings of previous studies that identified tumor size as a significant marker for higher-grade meningiomas [28–30]. Second, meningiomas with substantial peritumoral edema were often of a higher WHO grade, suggesting that the extent of edema could be indicative of tumor aggressiveness and poorer survival rates [16, 31–35]. Finally, elevated monocyte levels are significantly associated with high-grade meningiomas, underscoring the role of inflammation and the immune response in tumor pathology [36, 37].

These findings suggest potential clinical implications. Immunotherapies targeting immune checkpoints have shown promise in various cancers, with studies indicating increased PD-L1 expression on monocytes in high-grade meningiomas [38]. These findings suggest the potential integration of immune checkpoint inhibitors with conventional treatments to improve outcomes for patients with aggressive meningiomas [39].

The mainstay of meningioma treatment is surgical resection, which is complemented by radiation therapy when necessary. The importance of preoperative imaging and histopathological grading in guiding treatment strategies is well recognized [40]. The adoption of machine learning tools for tumor grading in imaging workflows promises to streamline clinical protocols, and the convergence of radiomics with genomic and other ‘omics’ technologies portends a shift toward precision medicine. For instance, research into the genetic underpinnings of meningiomas, particularly DNA methylation profiles, may yield new prognostic markers for tumor grading and recurrence [41–45].

However, this study is not without limitations. First, its retrospective design and the exclusion of cases with incomplete data may introduce selection bias. Second, the single-center context limits the generalizability of our findings, indicating the need for validation in a broader clinical setting. Third, the intricate relationship between patient outcomes and histological classification demands future research to incorporate prognostic data and enhance the ability to predict disease. Fourth, this study focused on developing models with high clinical interpretability to facilitate nomogram construction and clinical translation. Although we explored several classical machine learning models, more complex “black-box” models, such as random forest, XGBoost, or deep learning, were not included. Future research could further explore the potential and value of these advanced algorithms in meningioma grading prediction while ensuring model interpretability [46], such as incorporating explanatory tools such as SHAP. Finally, while the model’s predictive performance is commendable, direct biological and clinical interpretation of the selected high-dimensional radiomics features remains challenging. These features are mathematical abstractions derived from processed images, and their correlation with specific histopathological characteristics warrants further exploration.

## Conclusions

This study demonstrates that in the validation cohort, the radiomic model exhibited superior prognostic performance compared to both the clinical model and the integrated model. However, this finding may be attributed to the insufficient sample size. Before routine application, its true clinical utility needs to be validated through prospective multicenter studies.

## Supplementary Information

Below is the link to the electronic supplementary material.

Supplementary file1 (DOCX 13 KB)

## Data Availability

The datasets generated during and/or analyzed during the current study are available from the corresponding author on reasonable request.
