# Supplementary material for: A Novel Nomogram for Predicting Meningioma Grade Based on Radiomics Features and Clinical Characteristics
Source: Curr Med Sci. 2026 May 4;46(3):704–12. doi: 10.1007/s11596-026-00199-4 (PMC13315482; doi:10.1007/s11596-026-00199-4)
Supplement: Supplementary file 1 — Supplementary file1 (DOCX 13 KB) [file 11596_2026_199_MOESM1_ESM.docx]

**Table S1** Coefficients of selected radiomics features

| Feature | Coefficient |
| --- | --- |
| wavelet-LHL_glszm_GrayLevelNonUniformity | 0.09194446 |
| wavelet-HLL_glcm_Imc1 | 0.13047742 |
| wavelet-HHL_glszm_GrayLevelNonUniformity | 0.33845425 |
| wavelet-LLL_firstorder_Skewness | 0.05895908 |
